# Diversity of binary toxin positive *Clostridioides difficile* in Korea

**DOI:** 10.1038/s41598-023-27768-0

**Published:** 2023-01-11

**Authors:** Jieun Kim, Bongyoung Kim, Hyunjoo Pai

**Affiliations:** Division of Infectious Diseases, Department of Internal Medicine, Hanyang University College of Medicine, 222-1 Wangsimni-Ro, Seongdong-Gu, Seoul, 04763 Korea

**Keywords:** Microbiology, Medical research

## Abstract

The objective of this study is to determine the trend and diversity of binary toxin-positive *Clostridioides difficile* over 10 years in Korea. Binary toxin-positive strains were selected from a tertiary hospital in Korea in 2009–2018. The multi-locus sequence typing and antibiotic susceptibility test were performed. Among the 3278 isolates in 2009–2018, 58 possessed binary toxin genes (1.7%). The proportion of CDT- positive isolates was 0.51–4.82% in 2009–2018, which increased over the 10-year period (*P* = 0.023). Thirteen sequence types (STs) were identified; ST5 (14 [24%]), ST11 (11 [19%]), ST221 (10 [17%]), ST201 (7 [12%]) and ST1 (5 [9%]) were popular. All 58 isolates were susceptible to vancomycin and piperacillin/tazobactam, and clindamycin and moxifloxacin were active in 69.0% and 62% of isolates, respectively. ST1 strains were resistant to several antibiotics, including moxifloxacin (80%), clindamycin (60%) and rifaximin (60%). Moreover, four of five ST1 presented a metronidazole minimum inhibitory concentration of 4 µg/mL. Moxifloxacin resistance was highest (72.3%) for ST11. In conclusion, binary toxin-positive strains are non-prevalent in Korea and involve diverse STs. ST1 strains were resistant to several antibiotics.

## Introduction

*Clostridioides difficile* infection (CDI) is a common intestinal infection following disturbance of the gut microbiota, and mostly caused by antibiotic use. Over the last 10 years, binary toxin-positive strains of *C. difficile* have increased dramatically in North America and Europe, and epidemic strains have been identified as polymerase chain reaction (PCR) ribotype 027, REA group BI, and PFGE-type NAP1 (027/BI/NAP1)^1^. These strains produce binary toxin (CDT) in addition to toxin A (TcdA) and toxin B (TcdB), the main pathogenic toxins of *C. difficile*, and show other changes including fluoroquinolone resistance and 18-base pair deletion of the *tcdC* gene in the pathogenicity locus (PaLoc)^2^. Another important binary toxin-positive strains were identified in Netherland and other countries; the epidemic strains were PCR ribotype 078, REA group BK, and PFGE type NAP7 or NAP7,8 (078/BK/NAP7,8)^3^. The strains also produce binary toxins and have a deletion and stop codon in the *tcdC* gene^3^. Those epidemic binary toxin-positive strains are known to cause more severe infections with a poorer outcome, possibly because of virulence factors including binary toxins and hyperproduction of TcdA and TcdB due to deletion mutations in the *tcdC* gene^4^.

Besides these epidemic strains, other strains carry binary toxins whose clinical implications are not yet clear^5–7^. In the Asia–Pacific region, binary toxin-positive ribotype 027 strains are not prevalent in most countries, and epidemics of ribotype 027 or 078 have not been reported^8^. Likewise, an increase in CDI caused by binary toxin-positive strains has not been observed^8,9^, and clinical implication of binary-positive *C. difficile* isolates was not proved^9^.

In this study, to determine the trend and diversity of binary toxin-positive *C. difficile* over 10 years, we selected the CDT-positive isolates among all the isolates in 2009–2018 in one university hospital in Korea, and performed multi-locus sequence typing (MLST) and antibiotic susceptibility tests.

## Results

**Figure 1:**
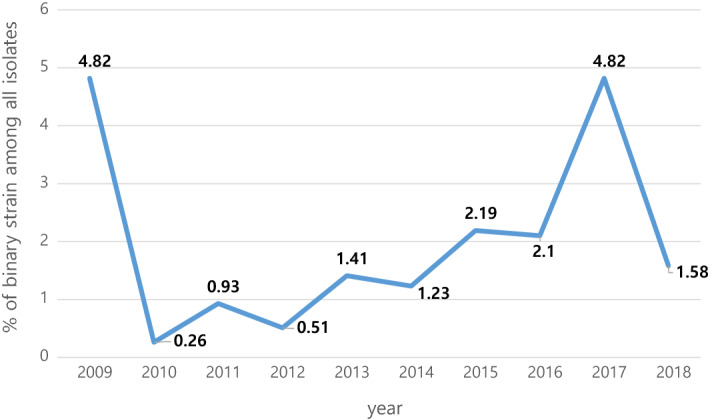
Annual percentage of binary toxin-positive *Clostridioides difficile* among all the isolates by year.

Among the 3278 isolates in 2009–2018, 58 possessed binary toxin genes (1.7%). One to twelve binary toxin-positive *C. difficile* strains were observed annually (Fig. 1). The prevalence of CDT- positive isolates was 0.51–4.82% in 2009–2018, and the proportion of binary toxin-positive isolates increased over the 10-year period (*P* = 0.023).

### MLST distribution and phylogenetic analysis

**Figure 2:**
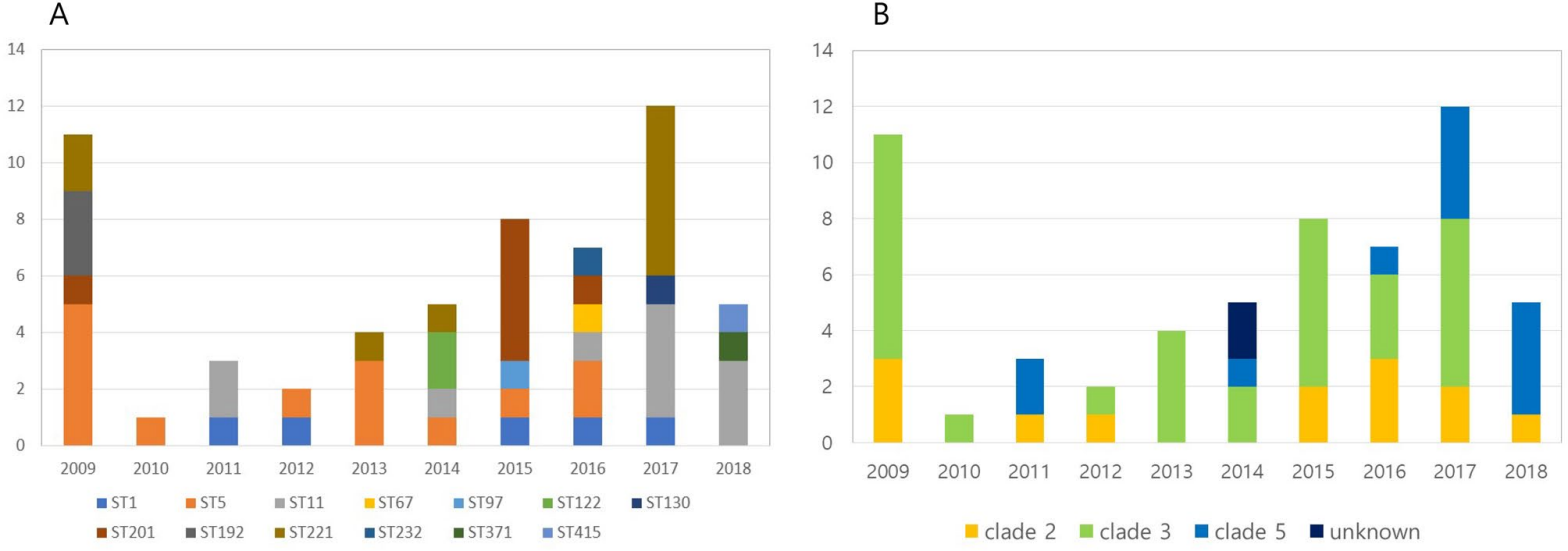
Distribution of (**A**) sequence types (ST) and (**B**) clades by year, 2009–2018.

All 13 STs typed in this study were compatible with other STs available in the online MLST *C. difficile* database. Among the 58 strains, 13 belonged to clade 2 (22.4%), 31 to clade 3 (53.5%), 12 to clade 5 (20.7%), and 2 to an unknown clade (3.5%). Thirteen STs were identified; ST5 (14 [24%]), ST11 (11 [19%]), ST221 (10 [17%]), ST201 (7 [12%]) and ST1 (5 [9%]) were popular strains (Table 1 and Fig. 2). In particular, ST11 strains appeared to have increased since 2011, and three of the five binary-producing strains in 2018 were ST11. However, the number of ST5 strains, most commonly found in 2009, decreased since 2013. ST1 had a similar annual incidence since its first identification in 2011.

**Table 1:** Antimicrobial susceptibility and geometric mean minimum inhibitory concentrations (µg/mL) of binary toxin–positive *Clostridioides difficile* isolates, 2009–2018.

clade	ST	n (%)	CLI			MXF			RFX		VAN		MTZ		TZP	
			%I (4)	%R (≥ 8)	GM MIC	%I (4)	%R (≥ 8)	GM MIC	%R (> 32)	GM MIC	%R (> 2)	GM MIC	%R (> 2)	GM MIC	%R (> 16/4)	GM MIC
2	1	5 (8.6)	0.0	60.0	32.0	0.0	80.0	16.0	60.0	5.3	0.0	1.3	80.0	2.6	0.0	16.0
	67	1 (1.7)	0.0	0.0	1.0	0.0	100.0	8.0	0.0	0.1	0.0	1.0	0.0	0.5	0.0	8.0
	97	1 (1.7)	0.0	0.0	0.5	0.0	0.0	2.0	0.0	0.1	0.0	1.0	0.0	1.0	0.0	16.0
	130	1 (1.7)	0.0	0.0	1.6	0.0	0.0	4.0	0.0	0.1	0.0	1.0	0.0	0.5	0.0	16.0
	192	3 (5.2)	0.0	0.0	2.3	100.0	0.0	5.3	0.0	0.2	0.0	1.2	0.0	0.9	0.0	13.9
	232	1 (1.7)	0.0	0.0	2.0	0.0	0.0	2.0	0.0	0.0	0.0	0.5	0.0	1.0	0.0	16.0
	371	1 (1.7)	0.0	100.0	32.0	100.0	0.0	4.0	0.0	0.0	0.0	1.0	0.0	1.0	0.0	16.0
3	5	14 (24.1)	7.1	0.0	0.6	7.1	0.0	2.1	0.0	0.1	0.0	1.0	0.0	0.6	0.0	15.2
	201	7 (15.5)	0.0	14.3	0.6	14.3	0.0	2.0	0.0	0.1	0.0	1.0	0.0	0.8	0.0	13.1
	221	10 (13.8)	0.0	10.0	0.8	10.0	0.0	2.1	10.0	0.1	0.0	0.9	0.0	0.3	0.0	14.9
5	11	11 (19.0)	54.6	27.3	9.7	0.0	72.7	11.0	9.1	0.1	0.0	0.6	0.0	0.4	0.0	16.0
	415	1 (1.7)	0.0	100.0	256.0	0.0	0.0	2.0	0.0	0.1	0.0	1.0	100.0	4.0	0.0	16.0
Un-known	122	2 (3.4)	0.0	50.0	2.8	0.0	100.0	32.0	0.0	0.1	0.0	0.7	0.0	0.5	0.0	11.3
Total		58	12.1	19.0		10.3	27.6		8.6		0.0		8.6		0.0	

*CLI*, clindamycin; *GM MIC*, geometric mean minimum inhibitory concentration; *I*, intermediate resistance; *MTZ*, metronidazole; *MXF*, moxifloxacin; *R*, resistance; *RFX*, rifaximin; *ST*, sequence types; *TZP*, piperacillin-tazobactam; *VAN*, vancomycin.

**Figure 3:**
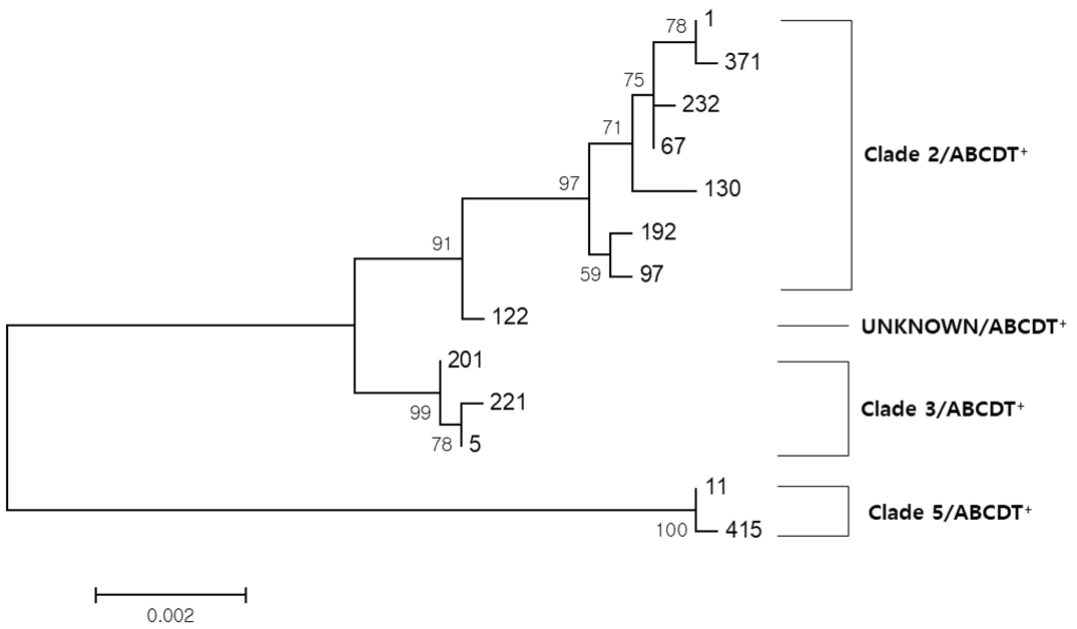
Neighbor-joining tree constructed using the concatenated sequences of the seven loci used in multi-locus sequence typing. Bootstraps were generated using 1000 replicates, and low values were removed for clarity. Sequence types (STs) are shown as numbers. The STs cluster into four groups, designated 2, 3, 5 and unknown.

Clade 2 contains the most diverse STs: ST1, five isolates; ST192, three isolates; ST67, ST97, ST130, ST232, ST371, one isolate each; and ST1 and ST192 were the most popular strains. Clade 3 was composed of three STs: ST5, 14 isolates; ST201, seven isolates; and ST221, 10 isolates. Among the isolates of clade 5, ST11 was the most common (11 isolates), and one ST415 strain was found. Two ST122 strains belonging to an unknown clade were identified in 2014 (Fig. 3).

A neighbor-joining tree was constructed using the concatenated sequences of the seven loci used in MLST (Fig. 3). STs were clustered into four groups, designated as 2, 3, 5, and undetermined. ST122 which was placed on the root of lineage 2, was designated as novel lineage 6 in a previous report^10^.

### Antibiotic susceptibility of binary toxin-positive *C. difficile* isolates

Table 1 presents the resistance rates of the 58 isolates to the six antibiotics. All isolates were susceptible to VAN and TZP, but CLI and MXF were active in 68.9% and 62.1% of the isolates, respectively. Five isolates (8.6%) were resistant to RFX and MTZ.

When we examined antibiotic susceptibility by ST type, ST1 strains showed a higher antibiotic resistance rate than other ST strains: MXF (80%), CLI (60%) and RFX (60%). Furthermore, 4 of 5 ST1 isolates presented an MTZ MIC of 4 µg/mL; thus the geometric mean MIC of MTZ was the highest among the ST1 isolates. One isolate of ST371, a strain closely related to ST1 according to MLST phylogeny, presented similar antibiotic susceptibility to ST1 (resistance [R] to CLI and intermediate resistance [IR] to MXF). The strains belonging to clade 3 did not present a high resistance rate to antibiotics: approximately 10% IR or R to CLI and approximately 10% IR to MXF. Among clade 5 strains composed of 11 ST11 and one ST415, MXF resistance was high (72.7%), but 27.3% and 9% of the isolates were resistant to CLI and RFX for ST11 strains. ST415 isolate had an MTZ MIC of 4 µg/mL. Two ST122 isolates belonging to a novel lineage showed 100% and 50% resistance to MXF and CLI, respectively.

## Discussion

Binary toxins (CDTs) belong to the family of binary ADP-ribosylating toxins consisting of two separate toxin components: CDTa, the enzymatic ADP-ribosyltransferase which modifies actin; and CDTb which binds to host cells and translocates CDTa into the cytosol. ADP-ribosylation induces depolymerization of the actin cytoskeleton, which produces membrane protrusions and aids bacterial adherence^2^. CDT production is associated with increased CDI severity^2,4,6,7^. Some binary toxin-positive strains have a *tcdC-*truncating mutation, a negative regulator of *tcdA* and *tcdB*, which may increase the infection severity^11^. However, truncating mutations in *tcdC* alone did not cause a difference in patient’ outcomes, although it was related to leukocytosis and C-reactive protein elevation^7^.

In this study, during the years of 2009–2018 at a university hospital in Korea, binary toxin-positive strains are not frequently encountered, and the epidemic binary-positive hypervirulent strains are not prevalent. Instead, ST-type strains in diverse clades existed in the hospital. However, ST11 strains showed a tendency to increase in this study, and similar findings were noticed in Taiwan and mainland China, but not in Japan^12–15^. High resistance to MXF of ST11 was commonly observed^12^, and high resistance to tigecycline was reported^13^. ST11/R078 strains began to be detected in 2011 and 2012 in the Asian countries^12^. R078 families are initially the predominant ribotype in production animals in the USA and Europe, and then in humans in Europe. Frequent presence of R078 in meat products suggests a possible transmission from animals to humans^16^. In terms of R027 isolates, they have not spread among Asian countries after the first introduction in Korea, Hong Kong and Australia in 2008–2010^17–19^. Despite hospital crowding, high levels of antibiotic (particularly fluoroquinolone) use, and huge aging population with multiple comorbidities, ST1 strains have not increased in these countries without clear explanation.

Binary-positive isolates from different clades showed different pattern of antibiotic susceptibility in this study. Despite a similar low prevalence, the epidemic hypervirulent ST1 and ST11 showed the higher resistance to most antibiotics. ST 371 most closely related to ST1 was resistant to MXF and CLI. Especially, ST1 showed 80% (4/5) of MTZ resistance despite MTZ resistance in *C. difficile* is rare. Those isolates showed the MIC of 4 µg/mL, and they were not considered from a single clone because all of them were from different years (1 isolate per year). It would be interesting to study the clonality and MTZ resistance mechanisms of those isolates.

MTZ resistance mechanisms in *C. difficile* are not clearly understood but are likely multifactorial processes involving alterations to metabolism such as with nitroreductase, iron uptake, DNA repair or biofilm formation^20,21^. Recently, MTZ resistance mediated by a high-copy number 7 kb plasmid (pCD-METRO) in *C. difficile* was discovered, which increased the MIC 25-fold^22^. The pCD-METRO was present in RT027 and non-toxigenic RT010 isolates from several countries. *C. difficile* RTs 027, 106, 001/072, 206, 010 and 356 strains had increased mean metronidazole MICs compared to other strains^20^.

Briefly reviewing the several binary-positive ST-types, all the ST-types below are positive for both toxin A and toxin B genes and the binary toxin genes. The ST67 strain, first reported in Japan did not contain an 18-base pair (bp) deletion or a one-bp deletion at position 117 in in *tcdC*, although it harbored eight nucleotide substitutions. The cytotoxicity of this strain was similar to that of ATCC BAA-1870 (RT027/ST1)^11^. ST5 and ST201 belonging to clade 3 have been sequenced in China^23^. The ST5 strain harbored a 54-bp consecutive deletion that resulted in a truncated TcdC protein, and ST201 also encodes a truncated TcdC. The ST122 strain, first isolated from Kuwait, contains an 18-bp deletion in the *tcdC* gene^10^. The phylogenetic analysis using a reference collection (Leeds-Leiden/ECDC) formed a well-separated sister clade to the clade formed by lineages 1 and 2, and the authors suggested a potential new lineage^10^. In our analysis, ST122 formed a separate lineage on the roots of lineage 2.

In summary, the proportion of binary toxin-positive strains was 1.7% among the 3278 isolates in 2009–2018. An epidemic strain of ST1 is not prevalent in Korea. The MLST analysis revealed diverse ST distributions in clades 2, 3, and 5. ST1 strains were more resistant to several antibiotics than other strains.

## Material and methods

### Study design and definition

This study was conducted at Hanyang University Hospital, a 900-bed tertiary care facility in Seoul, Korea. The study was approved by the institutional review board of Hanyang University Hospital (HYUH IRB 2016–01-031), which waived the need for informed consent.

All patients who had a CDI (as defined in the following paragraph) in 2009–2018 were identified through medical chart review, and the isolates from these patients were collected and stored. Diarrhea was defined as unformed stools more than three times per day on consecutive days or six times within 36 h^24^. The diagnosis of CDI was made using toxigenic culture, a commercial toxin A&B assay kit (VIDASⓇ *C. difficile* toxin A & B; BioMerieux SA, Marcy l’Etoile, France), and/or pseudomembrane on endoscopy^2^. Isolates from patients with CDI were tested by multiplex PCR^25^, and the isolates with CDT genes (*cdtA* and *cdtB*) were included.

### Isolation of *C. difficile* and detection of toxin genes by multiplex PCR

After alcohol shock treatment, stool specimens were cultivated on *C. difficile* moxalactam–norfloxacin–taurocholate agar (CDMN-TA agar; Oxoid Ltd., Cambridge, UK), supplemented with 7% horse blood^26^. Colonies of *C. difficile* were identified using Rapid ID 32A (BioMerieux SA). To identify the toxin genes, multiplex PCR was performed using template DNA, as described previously^25^. The positive controls were ATCC 43598 (PCR RT017), ATCC 9689 (PCR RT027), VPI 10643 (ATCC 43255, PCR RT087) and ATCC 700057, which represent A–B+CDT–, A+B+CDT+, A+B+CDT–, and A–B–CDT– RTs, respectively.

### Multi-locus sequence typing

Seven conserved housekeeping genes *adk, atpA, dxr, glyA, recA, sodA*, and *tpi* were sequenced^27^, and isolates were assigned to sequence types (STs) and clades in the *C. difficile* MLST database (https://pubmlst.org/organisms/clostridioides-difficile).

### Antibiotic susceptibility

The minimum inhibitory concentrations (MICs) of six antibiotics—metronidazole (MTZ), vancomycin (VAN), piperacillin/tazobactam (TZP), clindamycin (CLI), moxifloxacin (MXF) and rifaximin (RFX)—were determined. Brucella agar containing hemin (5 µg/mL), vitamin K1 (10 µg/mL), and 5% horse blood was used^28^. The MICs of CLI, MXF, and VAN were determined using Etest (AB-BIODISK, Solna, Sweden), while those of MTZ, RFX, and TZP were determined using the agar dilution test (Sigma-Aldrich, St. Louis, MO, USA). *C. difficile* ATCC 700057 was used as the control strain for the susceptibility tests. Resistance breakpoints were defined by the Clinical Laboratory and Standards Institute and European Committee on Antimicrobial Susceptibility Testing^28,29^.

### Phylogenetic analysis

A phylogenetic tree of the 13 STs was constructed using the seven housekeeping genes of the MLST. Each gene was separately aligned over 13 STs, and seven genes were concatenated for each ST. For the tree construction, the maximum likelihood method with the Tamura-Nei model was applied using MEGA6^30^. The robustness of the nodes was evaluated using the bootstrap method with 1000 replicates.

### Statistical methods

SPSS version 18.0 for Windows (SPSS Inc., Chicago, IL, USA) was used for the statistical analysis. A logistic regression analysis was performed, as appropriate. Statistical significance was set at *P* < 0.05.

### Ethics statement

The study was conducted according to the guidelines of the Declaration of Helsinki and approved by the Institutional Review Board of Hanyang University (protocol code 2016-01-031 and date of approval), and the requirement for written informed consent from patients was waived.

## Data Availability

The original contributions of this study are included in this article. Further enquiries can be directed to the corresponding author.
